# Communicating with Deaf Patients in the Clinical Environment: Lessons Learned from a Virtual Patient Panel

**DOI:** 10.12688/mep.20608.1

**Published:** 2024-10-11

**Authors:** Natalie P. Snyder, Benedicta O. Olonilua, Rosemary Frasso, Julia Croce, Dimitrios Papanagnou

**Affiliations:** 1 Thomas Jefferson University Sidney Kimmel Medical College, Philadelphia, Pennsylvania, USA; 2 Thomas Jefferson University College of Population Health, Philadelphia, Pennsylvania, USA; 3 Audiology, Thomas Jefferson University, Philadelphia, Pennsylvania, USA; 4 Emergency Medicine, Thomas Jefferson University, Philadelphia, Pennsylvania, USA

**Keywords:** Deaf, panel, communication, interpreter

## Abstract

**Background:**

Communication and cultural differences predispose Deaf patients to suboptimal healthcare. Despite this disparity, health professionals have historically received little to no training in caring for Deaf patients. Patient panels are an effective tool in medical education to model communication strategies.

**Objective:**

In this paper, we describe the design, implementation, and results of a virtual patient panel focused on communicating with Deaf patients in clinical contexts. We offer practical suggestions for incorporating similar educational interventions in health professions education to prepare trainees to effectively navigate these conversations with their patients.

**Methods:**

The panel consisted of a one-hour question and answer discussion facilitated by the authors with Deaf patients and Certified Deaf Interpreters (CDI). The panel was presented to 271 second-year medical students at our institution in November of 2023. Following this discussion, students were encouraged to share one or two key takeaways from the session through a survey link. These results were analyzed using pile-sorting qualitative analysis to identify main themes.

**Results:**

There were 73 respondents, with a response rate of 27%. After the panel, the most popular takeaway points from student reflections included communication ( *n*=56, 77%) and access to care ( *n*=47, 64%), followed by autonomy ( *n*=17, 23%), the doctor-patient relationship ( *n*=15, 21%), and culture ( *n*=11, 15%). Based on this initiative, we identified and offer twelve tips for developing similar exercises. These tips are thematically presented under three groupings: Planning Considerations, Patient Panelist Considerations, In-Session Considerations, and Post-Session Considerations.

**Conclusion:**

This patient panel was the first of its kind in our medical school curriculum. Important considerations in panel design and implementation should focus on delivery time constraints with live-interpreting and further exploring the role of trust and communication in the physician-patient relationship.

## Introduction

Communication and cultural differences predispose Deaf patients to suboptimal healthcare ^1^. Despite this disparity, health professionals have historically received little to no training in caring for Deaf patients ^2^. Patient panels are an effective tool in medical education to model communication strategies ^3^. We offer practical suggestions for incorporating similar educational interventions in health professions education to prepare trainees to effectively navigate these conversations with their patients.

In this paper, we describe the design, implementation, and results of a virtual patient panel focused on communicating with Deaf patients. This study is covered under a protocol that was reviewed and deemed exempt by the Thomas Jefferson University IRB under approval number 45 CFR 46.101 Control #22E.359 on April 28, 2022. Consent was waived by the IRB. The panel was a multidisciplinary collaboration between medical school curricular leadership, key Deaf stakeholders, and student advocates. The session consisted of a one-hour question and answer discussion facilitated by the authors with Deaf patients and Certified Deaf Interpreters (CDI) ^4^. The panel was presented to 271 second-year medical students at our institution as part of the Health Systems Science thread of the curriculum during their Neurology/Psychiatry organ-system block in November of 2023.

The panel began with a brief five-minute presentation from facilitators. First, there was an overview of the Americans with Disabilities Act (ADA) as it pertains to Deaf patients and their families ^5^. Students then learned the distinction between Big ‘D’ Deaf as a cultural identity and utilization of American Sign Language and little ‘d’ deaf as a state of hearing loss ^6, 7^. They also learned about the roles of various interpreters in healthcare settings, including certified Deaf interpreters (CDIs) who bridge cultural and linguistic gaps for culturally Deaf patients. Facilitators introduced the concept of Deaf-Hearing Team Interpreters, where CDIs, skilled in American Sign Language (ASL) and Deaf culture, collaborate closely with hearing interpreters to ensure accurate communication for Deaf patients. Subsequently, students engaged in a question-and-answer session with panelists, exploring topics such as when to request a CDI versus a hearing interpreter, common challenges when working with Deaf patients, and effective communication strategies in emergency situations without immediate interpreter availability. Following this discussion, students were encouraged to share one or two key takeaways from the session through a survey link. These results were analyzed using pile-sorting qualitative analysis to identify main themes.

There were 73 respondents, with a response rate of 27%. After the panel, the most popular takeaway points from student reflections included communication ( *n*=56, 77%) and access to care ( *n*=47, 64%), followed by autonomy ( *n*=17, 23%), the doctor-patient relationship ( *n*=15, 21%), and culture ( *n*=11, 15%). Many students highlighted novelty of the panel’s content, with one student describing Deaf-Hearing teams, stating, “I previously did not know about the process of Deaf interpretation with two interpreters to ensure complete and correct communication.” Another student reflected on “the big knowledge gap I had regard to caring for Deaf patients” prior to the panel.

Other students commented on the applicability of the panel for their future clinical practice. One student expressed, “I also recognize the value in having this session early in our education; giving our patients the confidence that they will receive the respect and proper communication to address their health needs is so important in maintaining a good patient-provider relationship.” Another student echoed this sentiment, saying “This is probably one of the most important patient panels we’ve had.” Similarly, one student emphasized, “I loved hearing about the different options we have for interpreters and types of interpretation services. This is good to know for clinical practice, to ensure we give the best available care to patients!”

## Twelve tips

Based on this initiative, we identified and offer twelve tips for developing similar exercises. These tips are thematically presented under three groupings: Planning Considerations, Patient Panelist Considerations, In-Session Considerations, and Post-Session Considerations.

*Planning considerations:*

1. **Identify the goals of the patient panel.** The goal of the panel was to recognize the role of the interpreter in the healthcare team. The gist of the panel is best encapsulated by one student’s takeaway: “Good communication requires patience and effort but is critical to patient care,” with another classmate stating, “We need to educate ourselves on what Deaf patients need and be prepared to advocate for them.” Another student’s reflection that “there’s a lot for us to learn about Deaf culture and about our patients who are Deaf – this was just an introduction, really!” highlights some of the complexities of distilling a large topic into a relatively short, focused session while also balancing the needs of all stakeholders involved (i.e., medical education curricular team, Deaf presenters, student advocates).
2. **Familiarize yourself with the technology.** Virtual patient panels have become increasingly popular considering the recent COVID-19 pandemic ^3^. It was important to consult with technological support regarding features of virtual platforms such as Zoom version 6.2.0 (40111) that would be compatible with showcasing interpreters during periods of signing. We also had to balance visual overstimulation of Deaf presenters, opting to show only the faces of our presenters and interpreters and not the audience. We chose the Zoom platform but opted not to record the session for privacy reasons. Chat features were enabled so that students could pose written questions in real-time.
3. ** Prepare facilitators for their assigned roles.** The first and second author of this work served as student advocates and facilitators of the panel. It was important to recognize dynamics between facilitators and Deaf panelists to manage time constraints, as there is a time lag between signing and spoken interpretation. The senior author of this work monitored the chat to organize and address student questions in real-time. Technological support staff provided expertise with screen-sharing and participant view.

*Patient panelist considerations:*

4. **Recruit a diverse panel.** It was crucial to recruit a diverse panel, including a Certified Deaf Interpreter (CDI) who also shared their perspective as a Deaf patient, and a hearing interpreter. This approach ensured a comprehensive understanding of the complexities involved in interpretation and communication for Deaf individuals within healthcare settings, as highlighted by the following student takeaways: “Interpretation can be complex and might require a team” and “I appreciated learning the nuances of etiquette and how to respond in various situations with Deaf patients. I also really appreciated having the perspective of interpreters.”
5. **Review the technology with panelists before the session.** Before the session, panelists were briefed on the operational details of Zoom for patient panels. They were informed that participants typically keep their cameras off and only turn them on to ask questions. To enhance clarity between Deaf patients and the interpreters, students should be instructed to type their questions in the chat instead of turning their camera and audio on to ask their question verbally. Moderators, with their cameras off, can then read these questions aloud during the session.
6. **Highlight functions of technology that will support panelists’ safety.** Ensuring the emotional support and comfort of our panelists, especially in a virtual setting, was paramount during our session. Recognizing the visual sensitivity of Deaf individuals who rely on ASL, we opted for a platform protocol where all student participants kept their cameras off and their microphones muted, except when asking questions. This approach aimed to minimize visual distractions and promote a focused environment for Deaf panelists and interpreters to effectively communicate. By prioritizing these accommodations, we aimed to create a supportive atmosphere while also facilitating meaningful interactions during our virtual patient panel.
7. ** Empower panelists to share a holistic version of themselves.** Panelists were encouraged to authentically share their perspectives, experiences, and challenges in healthcare settings. One panelist, for example, was a Deaf CDI who shared her experience as both a Deaf interpreter and a Deaf patient in healthcare settings. By empowering panelists to present a holistic version of themselves, medical students gained valuable insights into cultural nuances, communication preferences, and healthcare needs specific to the Deaf community. This approach fosters a deeper understanding among future physicians, as evidenced by the following student feedback: “It was very insightful to hear the voices of Deaf patients and [this experience] will shape my future encounters as a physician.”

*In-session considerations:*

8. ** Review online patient panel etiquette with students.** At the start of the session, we emphasized the importance of optimizing Zoom’s limited bandwidth for large meetings. To maintain clear communication, students were instructed to keep their cameras off and audio muted, allowing Deaf attendees to focus on the interpreters, who, along with the panelists, had their cameras on. This approach ensured an accessible and distraction-free environment for Deaf participants, enhancing their engagement and understanding during the panel discussion.
9. ** Start with a warm welcome and end with gratitude.** During the panel session, students were encouraged to briefly turn on their cameras at the beginning and end to allow panelists to see the audience they were engaging with and educating. This gesture not only showed respect and appreciation but also provided a visual connection that reinforced the impact of their insights and experiences shared during the session.
10. ** Use time efficiently.** During the session, we acknowledged that panelists had valuable stories to share within our allotted hour. To ensure their narratives were heard while staying on track, we informed them of our time constraints beforehand. We managed this by limiting the number of questions posed and gently guiding discussions back to the main topics when tangents arose, maintaining focus and respecting both their experiences and our session timeline.

*Post-session considerations:*

11. ** Debrief the session with your team.** After the panel session concluded, the organizers convened for a thorough debriefing. We discussed the overall flow of the session, considering how well it adhered to the goals of the session and time constraints. Emerging themes from the discussion were highlighted, including insights into Deaf patient perspectives and challenges in healthcare. We also reviewed student engagement, noting particularly insightful questions and responses. We discussed how well we managed to align discussions and questions to session objectives. Following this initial debriefing, which was conducted virtually over Zoom, we continued our discussions via email. This allowed us to delve deeper into our reflections on the session’s impact and to further refine our understanding of the panel’s outcomes. We exchanged thoughts on ways to enhance future panel sessions, considering both logistical aspects and content development to better serve our audience of medical students.
12. ** Express appreciation to panelists for participation.** After the session, panelists were promptly thanked and received words of appreciation in the Zoom chat from students. Additionally, during a subsequent debriefing afterward, we shared with panelists some of the insightful takeaways submitted by students, further expressing our gratitude for their participation and contributions. If budget allows, tokens of appreciation may also be considered (e.g., monetary honoraria, institutional swag, gift cards).

## Conclusions

This patient panel was the first of its kind in our medical school curriculum. Students emphasized themes of communication and access to care and highlighted the novelty and clinical applicability of the panel to their future practice. Important considerations in panel design and implementation should focus on delivery time constraints with live-interpreting and further exploring the role of trust and communication in the physician-patient relationship.

## Ethical approval and consent statement

This study is covered under the Thomas Jefferson University IRB approval number 45 CFR 46.101 Control #22E.359 on April 28, 2022. Consent was waived by the IRB. 

## Data Availability

Zenodo: Replication Data for: Communicating with deaf patients in the clinical environment: Lessons learned from a virtual patient panel. https://zenodo.org/doi/10.5281/zenodo.13830784 ^8^ This project contains the following underlying data: Data file 1. De-identified qualitative responses from participants sharing one or two key takeaways from the session. Data are available under the terms of the Creative Commons Zero “No rights reserved” data waiver (CC0 1.0 Public domain dedication).
