# Communicating with Deaf Patients in the Clinical Environment through Deaf-Hearing Teams: Lessons Learned from a Virtual Patient Panel

**DOI:** 10.12688/mep.20608.2

**Published:** 2025-02-05

**Authors:** Natalie P. Snyder, Benedicta O. Olonilua, Rosemary Frasso, Julia Croce, Dimitrios Papanagnou

**Affiliations:** 1 Thomas Jefferson University Sidney Kimmel Medical College, Philadelphia, Pennsylvania, USA; 2 Asano-Gonnella Center for Research in Medical Education and Healthcare, Thomas Jefferson University Sidney Kimmel Medical College, Philadelphia, Pennsylvania, USA; 3 Audiology, Thomas Jefferson University, Philadelphia, Pennsylvania, USA; 4 Emergency Medicine, Thomas Jefferson University, Philadelphia, Pennsylvania, USA

**Keywords:** Deaf, panel, communication, interpreter

## Abstract

**Background:**

Communication and cultural differences predispose Deaf patients to suboptimal healthcare. Despite this disparity, health professionals have historically received little to no training in caring for Deaf patients. Patient panels are an effective tool in medical education to model communication strategies.

**Objective:**

In this paper, we describe the design, implementation, and results of a virtual patient panel focused on communicating with Deaf patients in clinical contexts. We offer practical suggestions for incorporating similar educational interventions in health professions education to prepare trainees to effectively navigate these conversations with their patients.

**Methods:**

The panel consisted of a one-hour question and answer discussion facilitated by the authors with Deaf patients and Certified Deaf Interpreters (CDI). The panel was presented to 271 second-year medical students at our institution in November of 2023. Following this discussion, students were encouraged to share one or two key takeaways from the session through a survey link. These results were analyzed using pile-sorting qualitative analysis to identify main themes.

**Results:**

There were 73 respondents, with a response rate of 27%. After the panel, the most popular takeaway points from student reflections included communication ( *n*=56, 77%) and access to care ( *n*=47, 64%), followed by autonomy ( *n*=17, 23%), the doctor-patient relationship ( *n*=15, 21%), and culture ( *n*=11, 15%). Based on this initiative, we identified and offer twelve tips for developing similar exercises. These tips are thematically presented under three groupings: Virtual Panel Considerations, Curricular Considerations, and Special Considerations with Deaf Panelists.

**Conclusion:**

This patient panel was the first of its kind in our medical school curriculum. Important considerations in panel design and implementation should focus on delivery time constraints with live-interpreting and further exploring the role of trust and communication in the physician-patient relationship.

## Introduction

Communication and cultural differences predispose Deaf patients to suboptimal healthcare ^1^. Despite this disparity, health professionals have historically received little to no training in caring for Deaf patients ^2^. Patient panels are an effective tool in medical education to model communication strategies ^3^. We offer practical suggestions for incorporating similar educational interventions in health professions education to prepare trainees to effectively navigate these conversations with their patients.

In this paper, we describe the design, implementation, and results of a virtual patient panel focused on communicating with Deaf patients. This study is covered under a protocol that was reviewed and deemed exempt by the Thomas Jefferson University IRB under approval number 45 CFR 46.101 Control #22E.359 on April 28, 2022. Consent was waived by the IRB. The panel was a multidisciplinary collaboration between medical school curricular leadership, key Deaf stakeholders, and student advocates. The session consisted of a one-hour question and answer discussion facilitated by the authors with Deaf patients and Certified Deaf Interpreters (CDI) ^4^. The panel was presented to 271 second-year medical students at our institution as part of the Health Systems Science thread of the curriculum during their Neurology/Psychiatry organ-system block in November of 2023.

The panel began with a brief five-minute presentation from facilitators. First, there was an overview of the Americans with Disabilities Act (ADA) as it pertains to Deaf patients and their families ^5^. Students then learned the distinction between Big ‘D’ Deaf as a cultural identity and utilization of American Sign Language and little ‘d’ deaf as a state of hearing loss ^6, 7^. They also learned about the roles of various interpreters in healthcare settings, including certified Deaf interpreters (CDIs) who bridge cultural and linguistic gaps for culturally Deaf patients. Facilitators introduced the concept of Deaf-Hearing Team Interpreters, where CDIs, skilled in American Sign Language (ASL) and Deaf culture, collaborate closely with hearing interpreters to ensure accurate communication for Deaf patients. Subsequently, students engaged in a question-and-answer session with panelists, exploring topics such as when to request a CDI versus a hearing interpreter, common challenges when working with Deaf patients, and effective communication strategies in emergency situations without immediate interpreter availability. Following this discussion, students were encouraged to share one or two key takeaways from the session through a survey link. These results were analyzed using pile-sorting qualitative analysis to identify main themes.

There were 73 respondents, with a response rate of 27%. After the panel, the most popular takeaway points from student reflections included communication ( *n*=56, 77%) and access to care ( *n*=47, 64%), followed by autonomy ( *n*=17, 23%), the doctor-patient relationship ( *n*=15, 21%), and culture ( *n*=11, 15%). Many students highlighted novelty of the panel’s content, with one student describing Deaf-Hearing teams, stating, “I previously did not know about the process of Deaf interpretation with two interpreters to ensure complete and correct communication.” Another student reflected on “the big knowledge gap I had regard to caring for Deaf patients” prior to the panel.

Other students commented on the applicability of the panel for their future clinical practice. One student expressed, “I also recognize the value in having this session early in our education; giving our patients the confidence that they will receive the respect and proper communication to address their health needs is so important in maintaining a good patient-provider relationship.” Another student echoed this sentiment, saying “This is probably one of the most important patient panels we’ve had.” Similarly, one student emphasized, “I loved hearing about the different options we have for interpreters and types of interpretation services. This is good to know for clinical practice, to ensure we give the best available care to patients!”

## Twelve tips

Based on this initiative, we identified and offer twelve tips for developing similar exercises in a virtual setting. These tips are organized into three thematic categories: *Virtual Panel Considerations, Curricular Considerations, Special Considerations with Deaf Panelists*.

*Virtual Panel Considerations:*

1. **Familiarize yourself with the technology.** Virtual patient panels have become increasingly popular considering the recent COVID-19 pandemic ^3^. It was important to consult with technological support regarding features of virtual platforms such as Zoom (version 6.1.11 (17694)) that would be compatible with showcasing interpreters during periods of signing. We also had to balance visual overstimulation of Deaf presenters, opting to show only the faces of our presenters and interpreters and not the audience. We chose the Zoom platform but opted not to record the session for privacy reasons. Chat features were enabled so that students could pose written questions in real-time without utilizing spoken voice that may overwhelm interpreters and Deaf panelists.
2. **Start with a warm welcome and end with gratitude.** During the panel session, students were encouraged to briefly turn on their cameras at the beginning and end to allow panelists to see the audience they were engaging with and educating. This gesture not only showed respect and appreciation but also provided a visual connection that reinforced the impact of their insights and experiences shared during the session. After the session, panelists were promptly thanked and received words of appreciation in the Zoom chat from students. Additionally, during a subsequent debriefing afterward, we shared with panelists some of the insightful takeaways submitted by students, further expressing our gratitude for their participation and contributions. If budget allows, tokens of appreciation may also be considered (e.g., monetary honoraria, institutional swag, gift cards).
3. **Debrief the session with your team.** After the panel session concluded, the organizers convened for a thorough debriefing. We discussed the overall flow of the session, considering how well it adhered to the goals of the session and time constraints. Emerging themes from the discussion were highlighted, including insights into Deaf patient perspectives and challenges in healthcare. We also reviewed student engagement, noting particularly insightful questions and responses. We discussed how well we managed to align discussions and questions to session objectives. Following this initial debriefing, which was conducted virtually over Zoom, we continued our discussions via email. This allowed us to delve deeper into our reflections on the session’s impact and to further refine our understanding of the panel’s outcomes. We exchanged thoughts on ways to enhance future panel sessions, considering both logistical aspects and content development to better serve our audience of medical students.

*Curricular Considerations:*

4. **Identify the primary focus of the session.** It is difficult to distill such a complex subject – Deaf culture, the Deaf world view, treatment of d/Deaf patients in clinical settings – into a focused, 50-minute session. We had discussed different directions the panel could take, from Deaf patients sharing their experiences in healthcare to an introduction to Deaf culture and sensitivity training. While interesting, important, and novel to our students, these topics were too broad to fit in one patient panel. We ultimately decided to focus in on what resources students have available to them in working with d/Deaf patients. Through this approach, we could highlight skills that are relevant to students’ future clinical practice and begin to explore some of the nuances of Deaf culture. The gist of the panel is best encapsulated by one student’s takeaway: “We need to educate ourselves on what Deaf patients need and be prepared to advocate for them.” Another student’s reflection that “there’s a lot for us to learn about Deaf culture and about our patients who are Deaf – this was just an introduction, really!” highlights some of the complexities of distilling a large topic into a relatively short, focused session while also balancing the needs of all stakeholders involved (i.e., medical education curricular team, Deaf presenters, student advocates).
5. **Identify relevant Learning Objectives.** The goal of the panel was to introduce students to the concept of Certified Deaf Interpreters (CDI) and Deaf-Hearing interpreter teams. We had discussed how best to present this information, balancing the needs for didactic material for a brief introduction to enrich the discussion during the panelist question and answer portion. In developing these didactics, which would ultimately lead to discussion, our Learning Objectives were as follows:
  A. Explain the impact of the Americans with Disabilities Act (ADA) on providing comprehensive care for d/Deaf and hard of hearing patients.
    ▪  This is content that had been introduced and assessed earlier in the students’ preclinical curriculum. Given their foundational knowledge of this content, we opted for a quick refresher, emphasizing the role of the ADA with regards to d/Deaf patients and their rights to an interpreter.
  B. Discuss the dynamics of communicating with patients who are d/Deaf.
    ▪  We introduced the concept of Big ‘D’ Deaf vs little ‘d’ deaf, emphasizing the cultural, linguistic, and social identities inherent in Deafness. We also touched on various communication modalities, including American Sign Language and Computer Assisted Real-time Transcription (CART) and clinical situations in which these modalities can be used, from navigating the medical office space to explaining treatment plans.
  C. Recognize the interpreter’s role in the healthcare team.
    ▪  We concluded the didactic portion of the presentation with an introduction to Deaf-Hearing teams, explaining the role of the CDI as a cultural and linguistic expert and giving examples of when CDI teams may be especially useful (e.g., emergencies when unsure of d/Deaf patient’s ability to communicate; d/Deaf patients with language deprivation, other limited language skills, or other cognitive challenges; public broadcasts and news reports).
6. **Assessment of knowledge**. Our aim was twofold: first, we wanted to elicit feedback from the session to improve it for future iterations; second, we wanted to assess and reinforce students’ knowledge of these key concepts. To elicit feedback from students, we included a link where students could share free-form responses on some of their main takeaways from the session. This format allowed us to see a snapshot of students’ thoughts and if our intended Learning Objectives were well-received through this session. The Learning Objectives from this session were not assessed during or immediately after the session, however, students were assessed on this content as part of their cumulative exam for the curricular block.

*Special Considerations with Deaf Panelists:*

7. **Recruit a diverse panel.** It was crucial to recruit a diverse panel, including a Certified Deaf Interpreter (CDI) who also shared their perspective as a Deaf patient, and a hearing interpreter. Panelists were encouraged to authentically share their perspectives, experiences, and challenges in healthcare settings. By empowering panelists to present a holistic version of themselves, medical students gained valuable insights into cultural nuances, communication preferences, and healthcare needs specific to the Deaf community. This approach fosters a deeper understanding among future physicians, as evidenced by the following student feedback: “It was very insightful to hear the voices of Deaf patients and [this experience] will shape my future encounters as a physician.” Including a diversity of experiences and approaches was well-received by students, who reflected that “iInterpretation can be complex and might require a team” and “I appreciated learning the nuances of etiquette and how to respond in various situations with Deaf patients. I also really appreciated having the perspective of interpreters.”
8. **Utilize technology to support individuals’ visual preferences.** Recognizing the visual sensitivity of Deaf individuals who rely on ASL, we opted for a platform protocol where all student participants kept their cameras off and their microphones muted. To enhance clarity between Deaf patients and the interpreters, students should be instructed to type their questions in the chat instead of turning their camera and audio on to ask their question verbally. Moderators, with their cameras off, can then read these questions aloud during the session. This approach aimed to minimize visual distractions and promote a focused environment for Deaf panelists and interpreters to effectively communicate. By prioritizing these accommodations, we aimed to create a supportive atmosphere while also facilitating meaningful interactions during our virtual patient panel.
9. **Use time efficiently.** The first and second author of this work served as student advocates and facilitators of the panel. It was important to recognize dynamics between facilitators and Deaf panelists to manage time constraints, as there is a time lag between signing and spoken interpretation. The senior author of this work monitored the chat to organize and address student questions in real-time. Technological support staff provided expertise with screen-sharing and participant view. During the session, we acknowledged that panelists had valuable stories to share within our allotted fifty minutes. To ensure their narratives were heard while staying on track, we informed them of our time constraints beforehand. We managed this by limiting the number of questions posed and gently guiding discussions back to the main topics when tangents arose, maintaining focus and respecting both their experiences and our session timeline.
10. **Stakeholder engagement.** One of the most important considerations in coordinating this panel was managing the interests of multiple stakeholders, including Deaf professionals, medical school administration and educators, and student advocates. In our discussions about curricular development, we tried to strike a balance between our Deaf colleagues’ priorities and those of the medical school. There are many elements of Deaf culture and ASL that are unfamiliar to students, such as language deprivation resulting from inadequate childhood communication between deaf children and hearing family members. This significantly impacts literacy, socioemotional development, academic achievements, and health literacy ^8, 9^. Differences in communication and culture – and lack of healthcare provider awareness – have profound effects on d/Deaf people’s experiences in healthcare settings ^10, 11^. This background information sets the stage for the importance of CDIs as cultural mediators and how the development of one’s worldview can impact interactions with others.
11. **Student engagement.** This panel fell on the Tuesday before Thanksgiving break for students. We had discussed an in-person versus a virtual panel. In-person panels are more engaging, though we recognized that students may be resentful of a session right before their holiday break. We determined that students would be most receptive to a virtual session that would facilitate their travel during the busy holiday season. It is important to incentivize student involvement during the panel, especially since students are not used to having their cameras and microphones muted, which may prompt disengagement. Given the timing of this panel, students are only a few months away from taking Step 1 of their USMLE board exams and starting clinical rotations. Reminding students that this is an important opportunity to gain perspective before encountering these scenarios in practice helped facilitate student engagement in the chat.
12. **Community engagement.** We reminded students of our incredible community partners and the resources they have for furthering students’ education and exploration of American Sign Language and Deaf culture. Providing students with key contacts in the community allows for continued dialog and self-directed learning.

## Conclusions

This patient panel was the first of its kind in our medical school curriculum. Students emphasized themes of communication and access to care and highlighted the novelty and clinical applicability of the panel to their future practice. Important considerations in panel design and implementation should focus on delivery time constraints with live-interpreting and further exploring the role of trust and communication in the physician-patient relationship.

## Ethical approval and consent statement

This study is covered under the Thomas Jefferson University IRB approval number 45 CFR 46.101 Control #22E.359 on April 28, 2022. Consent was waived by the IRB. 

## Data Availability

Zenodo: Replication Data for: Communicating with deaf patients in the clinical environment: Lessons learned from a virtual patient panel. https://zenodo.org/doi/10.5281/zenodo.13830784 ^12^ This project contains the following underlying data: Data file 1. De-identified qualitative responses from participants sharing one or two key takeaways from the session. Data are available under the terms of the Creative Commons Zero “No rights reserved” data waiver (CC0 1.0 Public domain dedication).
