# Supplementary material for: Communicating with Deaf Patients in the Clinical Environment through Deaf-Hearing Teams: Lessons Learned from a Virtual Patient Panel
Source: MedEdPublish (2016). 2025 Feb 5;14:218. Originally published 2024 Oct 11. [Version 2] doi: 10.12688/mep.20608.2 (PMC11814955; doi:10.12688/mep.20608.2)
Supplement: Peer review history

# Reviewer report: Reviewer response for version 2

**DOI:** 10.21956/mep.22384.r41071

**Published:** 2025-03-10

**Authors:** Michael Cassara, Tabia Santos, Phillip Giarrusso

**Affiliations:** 1 Donald and Barbara Zucker School of Medicine at Hofstra/Northwell (Emergency Medicine, Science Education), Northwell Health (Ringgold ID: 5799), New Hyde Park, New York, USA; 2 Emergency Medicine, Northwell Health (Ringgold ID: 5799), New Hyde Park, New York, USA

Thank you for your submission to MedEdPublish. There are no actual or perceived conflicts of interest to report. 

**Reflexivity: **In full transparency, TS and PG (co-primary reviewers) are medical education and simulation fellows, respectively. Both have been mentored by MC (senior reviewer) throughout the entire peer review process. TS, PG, and MC are emergency physicians based at a single health system in the northeastern United States. In preparation for engaging the review process, TS, PG, and MC reviewed the MedEdPublish author guidelines for Practical Tips ( https://mededpublish.org/for-authors/article-guidelines/preparing-a-practical-tips-article)  and landmark articles on peer review and on health professions education scholarship/innovation. ^1-4^ We have also reviewed previous reviews. MC accepts full and ultimate responsibility for the content of the review. 

**Summary: **The authors’ manuscript describes twelve practical tips for the collaborative design, implementation, and outcomes measurement of a 60-minute session (brief 5-minute didactic review, see below, and a 50-minute virtual panel) for second-year medical students at a single institution in the United States. The goal of the session was to improve communication between future physicians and people who are d/Deaf (potentially future patients). The brief didactic was provided by faculty facilitators and focused on providing a quick review of the American Disabilities Act (its role and patients’ rights it guarantees; content was also previously covered in the curriculum),  distinction of the words “deaf” (state of hearing loss) and “Deaf” (cultural identity and/or utilization of American Sign Language or equivalent sign language), and the concept of Deaf-Hearing Team Interpreters and Certified Deaf Interpreters. The virtual panel focused on facilitators and barriers to effective communication between patients who are d/Deaf and healthcare team members; and, the interpreters’ roles when embedded within Deaf-hearing teams. The session was co-created with several key stakeholders: d/Deaf patients, medical student advocates, medical school faculty/curricular leadership, and interpreters. The session was situated within the “health systems science” thread of the curriculum during the “neurology/psychiatry” organ-system block (approximately 4 months into the second of a four-year program). Informed by their post-implementation debriefing and survey analysis, the authors’ propose twelve practical tips for educators interested in replicating this format at their institutions.

**Strengths: **The abstract is well written. The introduction appropriately establishes the relevance of the topic using Watling and Lingard’s “problem-gap-hook” heuristic. The authors frame their practical tips and advice across three domains: Virtual Panel Considerations, Curricular Considerations, Special Considerations with Deaf Panelists. We found this framing an effective way to present the tips. Most of the tips provide sound recommendations for readers seeking to replicate the same or similar instructional strategy for this subject matter (see limitations and recommendations). The manuscript is clear and easy to read in most areas with no obvious syntax or grammatical errors (see limitations). There are no figures, images, or tables. 

**Limitations: **The major limitations are the size of the  participant sample size and the low survey response rate, limiting the generalizability of findings related to the curriculum itself and, potentially by extension, the practical tips. The organization of some of the content in the introduction and in the Practical Tips section could be enhanced (see recommendations, below). 

**Recommendations/Suggested Revisions, by section**

**Title**

 1. The title was previously revised based on prior recommendations. We agree with the original recommendation to revise the original title. We feel the authors’ use of the term “Deaf-Hearing Teams” in the title remains slightly confusing, especially if readers are unfamiliar with this terminology. **Recommendation: Consider a slight adjustment to the title (“Communicating with Deaf Patients in the Clinical Environment through Deaf-Hearing Interpreter Teams”).** **Introduction**

 1. The authors clearly establish the significance of the problem of communication barriers between d/Deaf patients and healthcare providers and the importance of developing curricular innovations focused on overcoming them. Thank you.

 2. The first paragraph might benefit from additional explanation between the existing second and third sentences that describes what Deaf-Hearing Interpreter Teams are and demonstrates the gap in training among health professionals about how to work with them. **Recommendation: After the sentence beginning “Despite this disparity, health professionals…” consider adding 1-2 additional sentences describing what the Deaf-Hearing Interpreter Team is and why a two-interpreter approach should be taught for this specific population (see next comment).**

 3. In a few areas of the manuscript, the text appears misplaced and would benefit from a slight juxtapositional change. The Introduction is one area; the order of the last sentence in the first paragraph and the first sentence in the second paragraph should be inverted. **Recommendation: Consider adjusting the order of the last sentence in the first paragraph and the first sentence in the second paragraph and merge into one opening paragraph as follows:** " *Communication and cultural differences predispose Deaf patients to suboptimal healthcare. Despite this disparity, health professionals have historically received little to no training in caring for Deaf patients. [ MORE HERE ABOUT WHAT  DEAF-HEARING INTERPRETER TEAMS ARE, THE EXISTING PAUCITY OF TRAINING ABOUT THEM, AND WHY TRAINING ABOUT THE TWO-INTERPRETER APPROACH IS IMPORTANT ]  Patient panels are an effective tool in medical education to model communication strategies. In this paper, we describe the design, implementation, and results of a virtual patient panel focused on communicating with Deaf patients. We offer practical suggestions for incorporating similar educational interventions in health professions education to prepare trainees to effectively navigate these conversations with their patients.”*

 4. The section below this section would benefit from italicized subheaders (e.g., curriculum development, methods, and results, similar to the organization of the three domains of tips) to more clearly identify to the reader the parts of the curricular innovation in the introduction and better align with the abstract. Also, the manuscript is not reporting on a study per se, although I do acknowledge that the authors’ curriculum and survey procedures involved interaction and intervention with participants and required IRB review. **Recommendation (Consider headers as follows. In addition, consider replacing the word “study” (in the original Introduction, it is found in the second sentence of the second paragraph) with another word/phrase (e.g., “Our work”). See below:**​​​​

*Curriculum Development. Our work is covered under a protocol...etc.*

*Methods. The panel began with a brief five-minute presentation....etc.*

*Results. There were 73 respondents….etc.*

**Practical Tips | Virtual Panel Considerations.**

**Tip #1: **We found redundancy between this tip and tip #8 under the “Special Considerations” heading. We would prefer this section focus on tips to more effectively employ the technology supporting the virtual learning milieu. We felt that there are additional tips to include here, e.g. , how to pin selected speakers, closed captioning, live polling, tips on breakout use (if needed), and the need to dedicate at least one faculty member to monitor and facilitate the livestream chat. Also, how would the authors adjust these recommendations if the virtual panel were conducted using a different platform (i.e., MS Teams)? **Recommendation: Consider expanding this section based on the recommendations above, and keep discussion of how to optimize the experience to d/Deaf panelists to Tip #8 .**

**Curricular Considerations**

**Tip #4: **As this section is framed to focus specifically on “Curricular Considerations”, we were expected to see the well articulated goal in Tip #5 in this section. **Recommendation: Consider moving the sentence “The goal of the panel….” from Tip #5 to this section (Tip #4).**

**Tip #6. **We found this content well written and important to include, but this section is not written as a tip. **Recommendation: Revise the wording to give readers an actionable tip on how best to assess knowledge in this content domain and learning environment.**

**Special Considerations with Deaf Panelists**

**Tip #10. **As we read this tip in relation to the other tips in this section, we feel this should come earlier in sequence. **Recommendation: Consider reordering the sequence of tips in this section and place this one earlier.**

**Tip #11. **This tip is misplaced. Student engagement not a “Special Consideration.” **Recommendation: Move this tip under the “Curricular Considerations” section of the manuscript.**

**Other thoughts:**

- Has this curriculum been replicated elsewhere or reimplemented at the same institution since this implementation described in this manuscript? Data from another implementation could be added given the “living nature” of MedEdPublish articles and enhance the reporting of the innovation itself and the generalizability and validity of the Practical Tips that are offered. **Recommendation: Consider adding data from a second or third implementation to enhance generalizability. **

**Is there a clearly defined research question? No**

**Is there an adequate literature review that puts the question into context? Yes**

**Do the chosen methods match the question?  Yes**

**Are the results meaningful? No**

**Are the results translated and contextualized? Yes**

If evidence from practice is presented, are all the underlying source data available to ensure full reproducibility?

Yes

Is the topic of the practical tips discussed accurately in the context of the current literature

Yes

Are all factual statements correct and adequately supported by citations?

Yes

Are the conclusions drawn balanced and justified on the basis of the presented arguments?

Yes

Are arguments sufficiently supported by evidence from the published literature and/or the authors’ practice?

Partly

Reviewer Expertise:

health professions education, healthcare simulation, educational scholarship, educational innovation

We confirm that we have read this submission and believe that we have an appropriate level of expertise to confirm that it is of an acceptable scientific standard, however we have significant reservations, as outlined above.

## References

[1] Story, not study: 30 brief lessons to inspire health researchers as writers. Lingard, L., & Watling, C.(2021). *Cham: Springer International Publishing.* .

[2] The DoCTRINE guidelines: defined criteria to report innovations in education. Blanco, M., Prunuske, J., DiCorcia, M., Learman, L. A., Mutcheson, B., & Huang, G. C. *Academic Medicine* .(2022) 97(5), 689-695.;10.1097/ACM.000000000000463435171122

[3] METRICS: a pattern language of scholarship in medical education, Ellaway, R., & Topps, D. *MedEdPublish* .(2017). 6, 199.;10.15694/mep.2017.000199PMC1088522338406461

[4] Boyer's expanded definitions of scholarship, the standards for assessing scholarship, and the elusiveness of the scholarship of teaching,Glassick, C. E. *Academic Medicine* .(2000) 75(9), 877-880.;10.1097/00001888-200009000-0000710995607

# Reviewer report: Reviewer response for version 2

**DOI:** 10.21956/mep.22384.r41068

**Published:** 2025-02-13

**Authors:** Celia Hulme

**Affiliations:** 1 University of Manchester, Manchester, UK

**Whole manuscript:**

 It is always good to see new additions to literature regarding Deaf people and healthcare. Having read through the manuscript, there are areas that require some minor revision and please note that the comments below are not exhaustive of where areas can be amended.

 The manuscript requires several revisions to improve clarity, accuracy and focus. The title should be changed to better reflect the paper’s aim. The abstract needs rewording based on the changed in the main text. The introduction should expand on author positionality, panel structure and demographics while reducing redundant details. The Twelve Tips section should begin with an explanation of how the tips were developed, followed by actionable, standalone advice. Tips should be reorganised for logical flow, with clearer takeaways. The conclusion should summarise the tips’ value for educators.

***Title:***

 The title does not accurately reflect the objective of the paper. The paper does not focus on communicating with deaf patients in the clinical environment. Instead, it primarily provides tips on managing a virtual panel with Deaf sign language users, aimed at professionals delivering training in the healthcare sector. Therefore, the current title is misleading and confusing. A more suitable title might be ' *Lessons Learned from a Deaf Virtual Patient Panel: Best Practices for Engaging Deaf Patients in Clinical Contexts'*.

***Abstract***

 The abstract will need to be reworded to reflect the changes made in the main body. It should specify the country where this study is based and provide a more concise description of the type of Deaf individuals being referred to.

***Introduction:***

 The second paragraph in the introduction starting with ‘In this paper’, requires more information about the setup of the group. It would be beneficial to embed author positionality – providing backgrounds for each author and their involvement in this study. More details are needed about the key Deaf stakeholders and Deaf patients involved in the study are needed. Who are these stakeholders and how were they identified? What criteria were used to select the Deaf patients? How were they invited to participate? Demographics would be good to add here, particularly their language preferences (e.g. were all sign language users?). It would also be useful to provide a clearer breakdown of the panel. How many members were on the panel and what were their roles? How was the panel structured and how were the discussions facilitated? The panel was conducted virtually, specify the platform.  There is no definition of ‘Deaf’ provided in the paper. It would be helpful to clarify this term for readers who may be unfamiliar with its usage in this context. Mention that everyone was virtual because it reads as if the students were present in person and the panel was virtual.

 The third paragraph starting with ‘The panel began’, includes a level of detail about the content of the presentation that seems redundant, as it is covered in tip number 5. I would recommend reducing this section and expanding the second paragraph instead, as it requires more attention.

 The fourth paragraph starting with ‘There were 73 respondents’, raises a question about its relevance to the paper. This information might be more suitable for a different paper focusing on the impact of the presentation rather than how it was delivered. It does not align well with the overall aim of this paper. I would suggest removing it and instead briefly mentioning that a survey link was sent to students to gather their takeaway points. Similarly, the fifth paragraph starting with ‘Other students commented’ does not seem to add significant value to the paper. It would be summarised alongside the fourth paragraph, with a brief mention that students appreciated the training for its relevance to their future clinical practice.

***Twelve Tips:***

 At the start of this section, provide a detailed explanation used to identify the 12 tips, for example:

- Who was involved in creating these tips? Mention the roles of the team members.
- Was there a collaborative process?

A suggestion for each tip is that it should be framed as a standalone piece of advice that others can replicated. Use clear actionable language.

 Would curricular considerations be the more logical start than starting with Virtual Panel considerations?

**Virtual Panel Considerations**

 Tip 1: It would be useful to explain why Zoom was chosen over other platforms. Was it because it is more user friendly? Were other platforms considered, if so, why were they rejected?

 Tip 2: Would the debriefing part and the token of appreciation mention be better under the special considerations section (Tip 7)?

 Tip 3: Mention why there was a need for debriefing. Who was involved in the debriefing? Were there guiding/evaluation questions prepared before? What was learnt from this debriefing? What would be done differently next time? Clarify how student questions were recorded if the session was not recorded.  

**Curricular Considerations**

 Tip 4: Provide an actionable tip – what is the takeaway point here?

 Tip 5: What is the takeaway point here? What can readers learn from this?

 Tip 6: Clarify developed the survey questions? How many questions were asked? What is actionable from this tip?

**Special Considerations with Deaf Patients:**

 Tip 7: Explain why was it crucial to recruit a diverse panel.  It is not really clear who the panel is. As it reads, it was a CDI who is also a Deaf patient and a hearing interpreter.  How did the panelists share their experiences? Was it through a presentation or through Q and A? How were they selected to share their experiences during the session?

 Tip 8: This section is written well and clearly. Use this section as a model for other tips. It is concise, actionable and clearly communicates the takeway.

 Tip 9: You mention ‘the senior author’ – is this the first author? If so, reword with a more general description for all roles rather than authors.

 Tip 10: Rewrite to provide a clear actionable takeaway point.

 Tip 11: Fine

 Tip 12: This is an important tip –recognising community involvement. It would benefit from further elaboration. For instance, you could mention that community involvement provides cultural competence, suggests local Deaf organisations, offers accessible resources and facilitates ongoing interaction.

***Conclusion:***

 Remove references to students and focus on summarising the three main categories of tips, Highlight the value of the tips for readers i.e. the tips provide a practical framework and mention how educators can benefit from these tips.  

***Data availability:***

 If this section is not relevant to the paper, consider removing it.

If evidence from practice is presented, are all the underlying source data available to ensure full reproducibility?

Partly

Is the topic of the practical tips discussed accurately in the context of the current literature

Partly

Are all factual statements correct and adequately supported by citations?

Partly

Are the conclusions drawn balanced and justified on the basis of the presented arguments?

Partly

Are arguments sufficiently supported by evidence from the published literature and/or the authors’ practice?

Partly

Reviewer Expertise:

Sign language, Deaf people, healthcare contexts, mixed methods research, Lived Experience and Public Involvement.

I confirm that I have read this submission and believe that I have an appropriate level of expertise to confirm that it is of an acceptable scientific standard, however I have significant reservations, as outlined above.

# Reviewer report: Reviewer response for version 2

**DOI:** 10.21956/mep.22384.r41066

**Published:** 2025-02-11

**Authors:** Pamela Luft

**Affiliations:** 1 Kent State University, Kent, USA

The title revision is helpful and makes the focus of this article more clear.

 The revisions of the 12 tips also are much clearer and divide the key content into better-integrated categories. As the authors state, these provide much more detail now about the nature and extent of the efforts taken to prepare for, present, and follow up on this presentation. The additional comments from the students also provide more detail and support of the impacts this had on their preparation as future physicians. It would have been nice to include some of the final assessment data on this topic but the additional student comments are good evidence of their learning.

 Overall, the article provides more of the important details regarding critical issues that can serve as barriers to effective Deaf patient communication with health care providers.

If evidence from practice is presented, are all the underlying source data available to ensure full reproducibility?

Not applicable

Is the topic of the practical tips discussed accurately in the context of the current literature

Partly

Are all factual statements correct and adequately supported by citations?

Partly

Are the conclusions drawn balanced and justified on the basis of the presented arguments?

Partly

Are arguments sufficiently supported by evidence from the published literature and/or the authors’ practice?

Partly

Reviewer Expertise:

Deaf Education

I confirm that I have read this submission and believe that I have an appropriate level of expertise to confirm that it is of an acceptable scientific standard.

# Reviewer report: Reviewer response for version 1

**DOI:** 10.21956/mep.22061.r39794

**Published:** 2024-11-18

**Authors:** Pamela Luft

**Affiliations:** 1 Kent State University, Kent, USA

The title doesn't accurately reflects the focus of the manuscript which seems to be more about the 12 recommendations for doing a virtual patient panel than presenting specific “lessons learned” in preparing future health professionals to work with Deaf patients.

 Although the study purpose was to prepare future health practitioners to work with D/deaf individuals, the bulk of the manuscript’s content describes 12 tips for programs who wish to utilize video patient panels. Several of the 12 tips are somewhat redundant, perhaps because they are presented as a sequence of steps  rather than sets of recommended strategies. For example, three items address technology and two address appreciation and gratitude. Three other items could be considered as “preparation:” of facilitators, panelists, and students (regarding etiquette, etc.).

 The headings for the 12 tips are fairly generic and therefore, appropriate for all types of virtual panel presentations. Only within several of these are there specific recommendations regarding work with Deaf facilitators, interpreters, CDIs, and panelists. These unique aspects do not seem fully aligned to the otherwise general recommendations for implementing any type of virtual panel presentation. For example, the elements of Zoom utilized to support Deaf individuals’ visual preferences, the lag time between spoken or signed communication and interpretation which is increased when including a CDI would be more effective as a separate section of accommodations needed to ensure equal access, with a more explicit rationale and description of related issues.

 The first recommendation is to establish goals; yet, the goals for this panel did not address any of the key learning outcomes that would better prepare future health practitioners to work with D/deaf individuals. The stated goal was for students to “recognize” the role of the interpreters. But the respondents were only asked to provide “one or two key takeaways” that could be very general. This prompt addresses none of the content from the 5-minute presentation by facilitators that included the Americans with Disabilities Act, nor does it ask for important concepts regarding use of hearing interpreters and CDIs, lag time, visual accommodation needs with online platforms; or the unique language, communication, and world view differences including Deaf culture that were potentially evidenced by the Deaf panelists. The study went to some trouble to present and include these elements, but then required no evidence of having acquired this content from the students. This could have been done by a brief, online assessment that addressed this critical content. (This neglect of expecting retention of any key content potentially suggests a perspective that marginalizes Deaf individuals as individuals worthy of attention, which is quite common, but I believe this presentation was to help reduce. It currently appears that this content was not important enough to assess or to ask these health professionals to retain for their future practice.)

 Another important element that doesn’t appear to be present is with regard to Deaf individuals’ world view, that goes beyond Deaf culture. Many deaf children have inadequate childhood communication with family members that results in a lack of language fluency in ASL, English, or any other home language. This has significant impacts on their literacy, academic achievements, and access to world understandings despite having normal intelligence. This is the reason that CDI’s are important but this issue goes far beyond language use to include the individual’s perceptions of themselves, their needs, and the world in which they live. This can have substantial impacts on their interactions with health and other professionals such as profound conceptual misunderstandings and misinterpretations. The 12 suggestions are very appropriate for use by other programs planning to implement virtual patient panels.

 I suggest that the authors consider writing one article that suggests the strategies for generic virtual panel presentations, and another that addresses the unique elements for preparing, implementing, and concluding a virtual panel using Deaf patients. The latter should begin with a more extensive literature review of the struggles Deaf individuals have with accessing appropriate medical care due to the range of communication, world-view, and linguistic differences that many experience. The study’s preparation and content of the 5-minute overview also could be more fully described with an expanded rationale and recommendations for providing more extensive orientations, post-panel discussions, and assessments of students.

 This presentation took a very needed and important step in attempting to bridge the frequent communication and world-view differences that many Deaf individuals bring to healthcare settings. Overlooking or neglecting these issues often result in their further marginalization and misunderstandings that can substantially impact their healthcare treatments.

If evidence from practice is presented, are all the underlying source data available to ensure full reproducibility?

Not applicable

Is the topic of the practical tips discussed accurately in the context of the current literature

Partly

Are all factual statements correct and adequately supported by citations?

Partly

Are the conclusions drawn balanced and justified on the basis of the presented arguments?

Partly

Are arguments sufficiently supported by evidence from the published literature and/or the authors’ practice?

Partly

Reviewer Expertise:

Deaf Education

I confirm that I have read this submission and believe that I have an appropriate level of expertise to state that I do not consider it to be of an acceptable scientific standard, for reasons outlined above.

# Response

**Published:** 2025-01-24

**Authors:** Natalie Snyder

**Affiliations:** Thomas Jefferson University Sidney Kimmel Medical College, Philadelphia, Pennsylvania, USA

**Reviewer's comments:**

**The title doesn't accurately reflect the focus of the manuscript which seems to be more about the 12 recommendations for doing a virtual patient panel than presenting specific “lessons learned” in preparing future health professionals to work with Deaf patients.**

**Author's response:**

 We thank the reviewer for this point and agree that the title could be further refined to reflect this manuscript’s focus more accurately. In turn, we have updated the title: “Communicating with deaf patients in the clinical environment through deaf-hearing teams: Lessons learned from a virtual patient panel”.

**Reviewer's comments:**

**Although the study purpose was to prepare future health practitioners to work with D/deaf individuals, the bulk of the manuscript’s content describes 12 tips for programs who wish to utilize video patient panels. Several of the 12 tips are somewhat redundant, perhaps because they are presented as a sequence of steps rather than sets of recommended strategies. For example, three items address technology and two address appreciation and gratitude. Three other items could be considered as “preparation:” of facilitators, panelists, and students (regarding etiquette, etc.).**

**The headings for the 12 tips are fairly generic and therefore, appropriate for all types of virtual panel presentations. Only within several of these are there specific recommendations regarding work with Deaf facilitators, interpreters, CDIs, and panelists. These unique aspects do not seem fully aligned to the otherwise general recommendations for implementing any type of virtual panel presentation. For example, the elements of Zoom utilized to support Deaf individuals’ visual preferences, the lag time between spoken or signed communication and interpretation which is increased when including a CDI would be more effective as a separate section of accommodations needed to ensure equal access, with a more explicit rationale and description of related issues.**

**Author's response:**

 We thank the reviewer for these important observations. We have revised our 12 tips accordingly to reflect general considerations for a virtual panel, curricular considerations that showcase our thought process in the design of this panel, and special Deaf considerations that highlight the needs of this unique population.

**Reviewer's comments:**

**The first recommendation is to establish goals; yet the goals for this panel did not address any of the key learning outcomes that would better prepare future health practitioners to work with D/deaf individuals. The stated goal was for students to “recognize” the role of the interpreters. But the respondents were only asked to provide “one or two key takeaways” that could be very general. This prompt addresses none of the content from the 5-minute presentation by facilitators that included the Americans with Disabilities Act, nor does it ask for important concepts regarding use of hearing interpreters and CDIs, lag time, visual accommodation needs with online platforms; or the unique language, communication, and world view differences including Deaf culture that were potentially evidenced by the Deaf panelists. The study went to some trouble to present and include these elements but then required no evidence of having acquired this content from the students. This could have been done by a brief, online assessment that addressed this critical content. (This neglect of expecting retention of any key content potentially suggests a perspective that marginalizes Deaf individuals as individuals worthy of attention, which is quite common, but I believe this presentation was to help reduce. It currently appears that this content was not important enough to assess or to ask these health professionals to retain for their future practice.)**

**Author's response:**

 We appreciate the reviewer’s reflection on the data collection and assessment of student learning from this panel. We have updated the manuscript to reflect that this material, while not formally assessed during or immediately following the session, was formally tested as part of students’ cumulative final exams. We have also provided more of an explanation for why we elicited freeform responses and qualitative reflections from students after the session in lieu of a formal assessment.

**Reviewer's comments:**

**Another important element that doesn’t appear to be present is with regard to Deaf individuals’ world view, that goes beyond Deaf culture. Many deaf children have inadequate childhood communication with family members that results in a lack of language fluency in ASL, English, or any other home language. This has significant impacts on their literacy, academic achievements, and access to world understandings despite having normal intelligence. This is the reason that CDI’s are important but this issue goes far beyond language use to include the individual’s perceptions of themselves, their needs, and the world in which they live. This can have substantial impacts on their interactions with health and other professionals such as profound conceptual misunderstandings and misinterpretations. The 12 suggestions are very appropriate for use by other programs planning to implement virtual patient panels.**

**Author's response:**

 This is an incredibly important and interesting point. While this is of great interest to the authors, it was not the primary goal of the panel or the manuscript. We have included some more of this information in the current revision to help contextualize the importance of CDIs – which was the primary focus of this work. We thank the reviewer for this comment and allowing us the opportunity to provide some more background information.

**Reviewer's comments:**

**I suggest that the authors consider writing one article that suggests the strategies for generic virtual panel presentations, and another that addresses the unique elements for preparing, implementing, and concluding a virtual panel using Deaf patients. The latter should begin with a more extensive literature review of the struggles Deaf individuals have with accessing appropriate medical care due to the range of communication, world-view, and linguistic differences that many experience. The study’s preparation and content of the 5-minute overview also could be more fully described with an expanded rationale and recommendations for providing more extensive orientations, post-panel discussions, and assessments of students.**

**Author's response:**

 We thank the reviewer for this suggestion. The senior author of this work has already published extensively on strategies for generic virtual panel presentations (and his work is cited in the present study). As mentioned above, we have decided to revise the 12 tips to incorporate some best practices for virtual panels in general ( *Virtual Panel Considerations*) and then dedicate the bulk of the tips to curricular development and panelist needs ( *Curricular Considerations* and *Special Considerations with Deaf Panelists*, respectively). We hope that with this revision, the manuscript more directly addresses strategies for replicating this type of work.

**Reviewer's comments:**

**This presentation took a very needed and important step in attempting to bridge the frequent communication and world-view differences that many Deaf individuals bring to healthcare settings. Overlooking or neglecting these issues often result in their further marginalization and misunderstandings that can substantially impact their healthcare treatments.**

**Author's response:**

 We appreciate the reviewer’s thoughts and reflections. We agree that this is an important area for study and innovation and are honored to take steps to address communication and cultural misunderstandings.

# Reviewer report: Reviewer response for version 1

**DOI:** 10.21956/mep.22061.r39601

**Published:** 2024-11-08

**Authors:** Mrutyunjaya Mishra

**Affiliations:** 1 Dr. Shakuntala Misra National Rehabilitation University, Lucknow, India

The research carried out is needed for providing clinical services to the Deaf as well as Hard of Hearing (DHH) population. However technological developments have made it easier to communicate with the DHH population. There are some applications like SignAble (https://play.google.com/store/apps/details?id=com.signable.user&pcampaignid=web_share) helping the Deaf population in consulting the clinical professionals. However sensitising the medical professionals about the need and supports required will be beneficial. short term courses in Sign Language (SL) for medical practitioner will also be helpful to deal with DHH patients. it should be included in the curriculum of different courses of professional study to facilitate communication between the professionals and DHH  population.

 the research study used the virtual panel in which communication, access, autonomy , relationship between doctor and patients as well as cultural aspects is novel and the result is presented through planning consideration, patient panelist consideration, in-session consideration and post- session consideration is presented well.

 The study included 73 respondents which may be conducted in different geographical areas for standardisation.

 The tips given will be helpful for future researches in this field.

 I applaud the efforts taken by the researchers and wish them good luck for conducting such researches in the future for the inclusion of DHH population.

If evidence from practice is presented, are all the underlying source data available to ensure full reproducibility?

Yes

Is the topic of the practical tips discussed accurately in the context of the current literature

Yes

Are all factual statements correct and adequately supported by citations?

Yes

Are the conclusions drawn balanced and justified on the basis of the presented arguments?

Yes

Are arguments sufficiently supported by evidence from the published literature and/or the authors’ practice?

Yes

Reviewer Expertise:

i am working in the area of hearing impairment. presently i am associate professor in the department of hearing impairment in Dr. Shakuntala Misra National Rehabilitation University, Lucknow (https://dsmru.up.nic.in/) . I am also involved in various researches in sign language and neurological aspects of Deaf population.

I confirm that I have read this submission and believe that I have an appropriate level of expertise to confirm that it is of an acceptable scientific standard.

## References

[1] *SignAble* . Reference source
